# Geriatric Nutritional Risk Index and First-Year Mortality in Incident Hemodialysis Patients

**DOI:** 10.3390/nu16050652

**Published:** 2024-02-26

**Authors:** Gi Hyun Song, Han Byul Choi, Hayne Cho Park, Do Hyoung Kim, Young-Ki Lee, AJin Cho

**Affiliations:** 1 Department of Internal Medicine, Kangnam Sacred Heart Hospital, Seoul 07441, Republic of Korea; khsong0912@gmail.com (G.H.S.); starfactory@daum.net (H.B.C.); haynepark798@hallym.or.kr (H.C.P.); dhkim6489@hallym.or.kr (D.H.K.); km2071@hallym.or.kr (Y.-K.L.); 2 Kidney Research Institute, Hallym University, Seoul 07441, Republic of Korea

**Keywords:** Geriatric Nutritional Risk Index, nutrition, hemodialysis, mortality

## Abstract

Objective. The Geriatric Nutritional Risk Index is a simple nutritional screening method, and this study aimed to investigate the association between the initial Geriatric Nutritional Risk Index and all-cause mortality in incident patients in the first year after the initiation of hemodialysis. Materials and Methods. This study is a retrospective cohort study and used the Korean Renal Data System database. Patients who were eligible for Geriatric Nutritional Risk Index assessment and underwent hemodialysis from January 2016 to December 2019 were included. The primary outcome was all-cause mortality, and outcome evaluation was performed in December 2020. A Cox proportional hazard model was used to analyze the association between the Geriatric Nutritional Risk Index and mortality. Results. A total of 10,545 patients were included, and the mean age was 63.9 ± 3.7 years. The patients were divided into four groups by the quartile of the Geriatric Nutritional Risk Index with a mean value of 96.2 ± 8.2. During the study period, 545 (5.2%) deaths occurred. The surviving patients had higher Geriatric Nutritional Risk Index values than ones who died in the first year of hemodialysis initiation (96.6 ± 7.5 vs. 88.2 ± 9.3, *p* < 0.001). Quartile 1 (Geriatric Nutritional Risk Index < 91.8) showed a significantly increased risk of all-cause (Hazard Ratio: 2.56; 95% Confidence Interval: 2.13–3.09; *p* < 0.001) and cardiovascular mortality (Hazard Ratio: 22.29; 95% Confidence Interval: 1.71– 3.08; *p* < 0.001) at the first year in comparison with Quartile 4 (Geriatric Nutritional Risk Index ≥ 101.3). In areas under the receiver-operating characteristic curves of all-cause mortality, the Geriatric Nutritional Risk Index model improved predictive values, compared to the baseline model. The area with the Geriatric Nutritional Risk Index model was significantly higher than the one with a model including albumin or body mass index (*p* < 0.001). Conclusions. These findings suggest that a low Geriatric Nutritional Risk Index (<91.8) is associated with first-year all-cause and cardiovascular mortality in patients who start hemodialysis and may be a useful and reproducible tool for assessing prognoses in this population.

## 1. Introduction

Patients with end-stage renal disease (ESRD) and on hemodialysis (HD) have a higher mortality rate than the general population. Notably, a retrospective cohort study of the early mortality among incident HD patients showed an increased risk of death during the first 120 days compared with the 121–365 days after the initiation of HD [1,2]. A recent cohort study found that the mortality rate of patients with ESRD was 23.9% for the first 90 days and 19.3% for 1 year after starting HD [3]. Therefore, focused attempts to improve HD mortality outcomes in the first few months of dialysis are important in patients initiating HD.

Even though previous studies identified several important factors associated with elevated mortality among incident HD patients, few have revealed malnutrition as a risk factor for early mortality in dialysis [2,4,5,6]. The nutritional problem is one of the modifiable associating factors for mortality in patients with ESRD and a simple and appropriate method could help identify HD patients at a high risk for malnutrition. Many nutritional screening tools have been developed for general purposes and were further adapted for nutritional assessment in HD patients [7,8,9]. Among them, the Geriatric Nutritional Risk Index (GNRI) is recommended as a simple method for nutritional assessment; GNRI utilizes only three objective parameters, namely body weight, height, and serum albumin [10]. A study of the usefulness of several available nutritional tools indicated GNRI as the simplest and most accurate risk index for identifying maintenance HD patients with malnutrition [11].

GNRI has been known as a prognostic factor for all-cause and cardiovascular mortality [10,12] of HD patients. Our previous study demonstrated that low GNRI in a negative trend is associated with increased mortality in HD patients [13]. In previous studies, a GNRI less than 91.2 indicated a high possibility of malnutrition and is associated with all-cause mortality [11,14]. However, the GNRI range related with a poor prognosis is not well known in patients undergoing HD. There are few studies about the association between GNRI at initiation and early mortality in HD patients. GNRI is a simple indicator of the nutritional status and prognosis, so if it is associated with high mortality early after HD, it may be helpful to improve patient outcomes. In this study, we aimed to test whether the initial baseline GNRI value affects early mortality for up to 1 year after the initiation of HD, and assessed which range of GNRI is associated with early mortality from the Korean Renal Data System (KORDS).

## 2. Materials and Methods

### 2.1. Study Design and Participants

The Korean Society of Nephrology (KSN) has maintained KORDS, a nationwide ESRD registry, which has continued for over 35 years. The online registry program on the KSN website has been administered since 2001 and the KORDS Committee has collected data about dialysis centers and patients with ESRD via this program (http://www.ksn.or.kr, accessed on 30 October 2022). Physicians in each dialysis center have updated the data based on individual medical records. The registry is not dependent on patients’ insurance status. Details of the methods have been described elsewhere [15,16]. For this study, we examined the cohort of all incident HD patients in the KORDS database who started HD from 2016 to December 2019. There is a total of 29,098 patients with ESRD (age ≥ 18 years) who started HD and were registered in KORDS in this period. Among them, 10,545 patients were eligible for initial GNRI assessment and were included in this study. In December 2020, study outcomes were evaluated. This study was conducted according to the Ethics of Clinical Research (Declaration of Helsinki) and was approved by the Institutional Review Board (IRB) of Hallym University Kangnam Sacred Heart Hospital, Seoul, South Korea (IRB No.: 2021-04-036). Due to the use of de-identified retrospective data, the IRB waived the requirement for written patient consent.

### 2.2. Demographic and Clinical Data

Demographic and clinical data (main causes of ESRD, comorbidities, and vascular access type) were collected within 1 month after HD initiation. For each patient, we obtained data on laboratory parameters prior to HD, including serum albumin, hemoglobin, phosphorus, calcium, intact parathyroid hormone (PTH), and cholesterol. Kt/V is single-pool Kt/V and means the HD dose. The main causes of ESRD included diabetes, hypertension, glomerulonephritis, and others. Cardiovascular comorbidities included coronary artery disease, heart failure, arrhythmias, and cerebrovascular disease. Cerebrovascular disease refers to stroke and peripheral vascular disease. We also looked at chronic obstructive pulmonary disease (COPD), liver disease, and gastrointestinal disease (GI). Liver disease included hepatitis B and C and liver cirrhosis. GI disease included gastric and duodenal ulcers. Vascular access was recorded as the use of an arteriovenous fistula, arteriovenous graft, or catheter.

### 2.3. GNRI Calculation and Outcomes

GNRI was calculated based on the following formula: [1.489 × albumin (g/L)] + [41.7 × (dry weight/ideal weight)] [17]. When the dry weight was greater than the ideal weight, weight/ideal weight was set at 1. The ideal weight was calculated using height and a BMI of 22 kg/m^2^, which is associated with the lowest mortality and validated [18]. Patients were divided into four groups by GNRI quartiles (Quartile 1: 56.7–91.7; Quartile 2: 91.8–97.1; Quartile 3: 97.2–101.2; Quartile 4: 101.3–129.6). 

The primary outcome of interest was all-cause mortality during the first year of HD treatment. Therefore, patients were followed until the end of this study, change to peritoneal dialysis (PD), or death. The secondary outcome was cardiovascular mortality, which is death caused by cardiovascular events, namely coronary artery disease, sudden cardiac arrest, stroke, and pulmonary embolism.

### 2.4. Statistical Analysis

The baseline patients’ characteristics were compared by GNRI quartiles, and the percentages for categorical variables and the mean ± standard deviation for continuous variables were calculated. The groups were compared using an analysis of variance and chi-squared test. We examined the distribution of GNRIs in patients who died and those who survived to day 120 and to days 121–365 after HD initiation and used t-tests to compare the two groups at each time point. We analyzed the survival rate between GNRI quartiles by the Kaplan–Meier method, and compared the difference using the log-rank test. A Cox proportional regression model was used to analyze the association between the GNRI quartiles and mortality in the first year after HD initiation. We first analyzed the univariate risk of all-cause and cardiovascular mortality from Quartile 1 to 3 using Quartile 4 as the reference group. In the multivariate model, we adjusted for the age, sex, main cause of ESRD, systolic blood pressure, diastolic blood pressure, presence of comorbidities, vascular access, and laboratory findings. There are missing values for 104 patients. We performed complete case analyses in the multivariate analyses. The proportional hazards’ assumption was tested using the Schoenfeld residual method. An accuracy comparison of the models for all-cause and cardiovascular mortality including GNRI or BMI or albumin was analyzed by receiver-operating characteristic (ROC) curves. The difference of Area Under the ROC Curve (AUC) values of the baseline model and model with GNRI or BMI or albumin was tested using the DeLong test. We compared AUC values between models using GNRI and models using BMI or albumin. All *p*-values involved two-sided tests, and *p* < 0.05 was considered statistically significant. R version 4.0.5 (R Foundation for Statistical Computing, Vienna, Austria) was used to analyze the data.

## 3. Results

### 3.1. Baseline Characteristics of the Patients

Table 1 shows the clinical and laboratory characteristics of the patients. The mean age was 63.9 ± 13.7 years and 37.2% were women. There were 53% of patients with diabetes as a main cause of ESRD and 66.5% of the patients used an arteriovenous fistula (AVF) for vascular access. The mean GNRI was 96.2 ± 8.2. Patients with lower GNRI levels at HD initiation were older and more likely to be female. The group with GNRI < 91.8 (Quartile 1) had a higher proportion of heart failure, cerebrovascular disease, COPD, and liver disease, and a lower proportion of AVF and lower serum hemoglobin, calcium, and phosphorus values. Those in the top quartile were younger, more likely to be male, and less likely to have cardiovascular complications, liver disease, or GI disease. They were also more likely to use an AVF for vascular access and had higher hemoglobin, calcium, and phosphorus concentrations in blood tests.

### 3.2. GNRI Distribution

**Figure 1:**
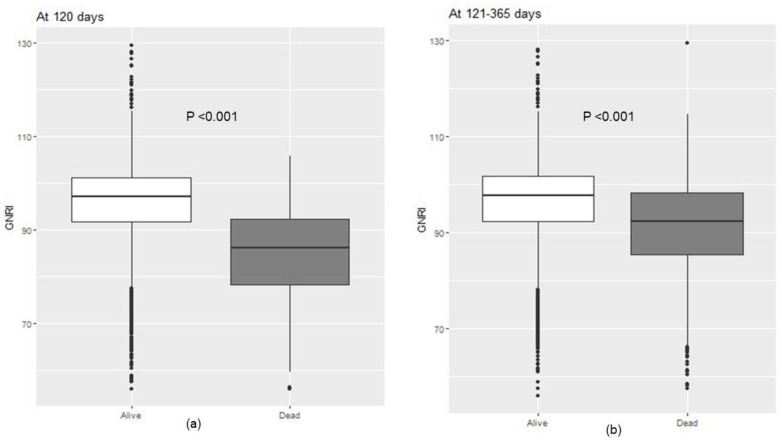
Box plots of GNRI values at 120 (**a**) and 121–365 (**b**) days in study population. Abbreviation: GNRI, Geriatric Nutritional Risk Index.

The GNRI distribution by the survival status is shown in Figure 1. The surviving patients had higher GNRI values than those who died during the first year after HD initiation (96.6 ± 7.5 vs. 88.2 ± 9.3, *p* < 0.001). At less than 120 days, the mean value of GNRIs was 85.5 ± 12.3 for dead patients and 96.4 ± 9.4 for survivors (*p* < 0.001). At 121–365 days, the values were also higher for survivors (96.9 ± 4.7 vs. 91.5 ± 6.0, *p* < 0.001), but the GNRI difference by the survival status became smaller.

### 3.3. Association between GNRI and First-Year Mortality

**Figure 2:**
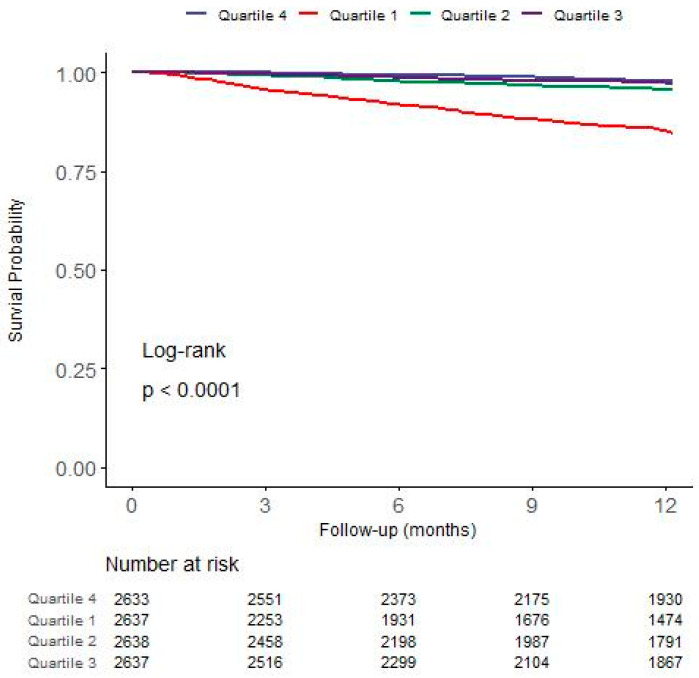
Kaplan–Meier curves of GNRI groups for all-cause mortality. Abbreviation: GNRI, Geriatric Nutritional Risk Index.

The group with GNRI < 91.8 (Quartile 1) showed the lowest survival in the first year after HD initiation (*p* < 0.001; Figure 2). Seven patients were transferred to PD, and 545 (5.2%) deaths occurred during the study period. At 365 days, the survival rates were 85.1%, 95.7%, 97.3%, and 97.7% for Quartile 1, Quartile 2, Quartile 3, and Quartile 4, respectively. We performed Cox regression analyses to assess the effects of GNRI on first-year mortality in incident HD patients (Table 2). In the univariate model, patients in Quartile 1 (Hazard Ratio (HR): 7.74; 95% Confidence Interval (CI): 5.76–10.36; *p* < 0.001) and Quartile 2 (HR: 2.08; 95%CI: 1.48–291; *p* < 0.001) had a higher mortality rate compared to patients in Quartile 4. Patients in Quartile 1 also had significantly higher cardiovascular disease mortality compared to patients in Quartile 4 (HR: 6.22; 95% CI: 4.07–9.50; *p* < 0.001). After adjusting for the age, sex, main cause of ESRD, systolic blood pressure, diastolic blood pressure, presence of comorbidities, vascular access, and laboratory findings, Quartile 1 showed a significantly increased all-cause mortality (HR: 2.56; 95% CI: 2.13–3.09; *p* < 0.001) in comparison with Quartile 4. The risk of cardiovascular mortality was also high in Quartile 1 (HR: 2.29; 95% CI: 1.71–3.08; *p* < 0.001). 

### 3.4. Comparison of Predictive Values for Mortality

We compared the predictive values from models including BMI or albumin or GNRI for all-cause and cardiovascular mortality (Table 3). The baseline model included age, sex, main causes of ESRD, systolic blood pressure, diastolic blood pressure, the presence of comorbidities, vascular access, and laboratory findings. The AUC value of the baseline model was 70.8 (68.4–73.2) for all-cause mortality and 70.1 (66.4–73.8) for cardiovascular mortality. The addition of BMI or albumin or GNRI to the baseline model significantly increased AUC for predicting all-cause mortality. The AUC for all-cause mortality was significantly different between models with GNRI and albumin (*p* = 0.009) or BMI (*p* < 0.001). Adding albumin or GNRI was significantly more favorable than the baseline model for predicting cardiovascular mortality, with the GNRI model possessing a higher AUC than the BMI model (*p* < 0.001).

### 3.5. Subgroup Analyses for Mortality

Table 4 represents subgroup analyses for all-cause mortality. In most groups, the low GNRI was consistently associated with increased mortality. The age, sex, diabetes status, vascular access type, history of cardiovascular disease, anemia, and phosphorus concentration did not affect the finding that low GNRI levels were associated with increased all-cause mortality.

## 4. Discussion

This study examined the association between GNRI and all-cause and cardiovascular mortality in incident HD patients in the first year after HD initiation. GNRI values in patients alive at 120 days and during days 121–365 were higher than in those who died within the corresponding periods. Furthermore, GNRI <91.8 was an independent risk factor for all-cause and cardiovascular mortality in this population. When we compared the predictive accuracy of all-cause mortality between the models, the GNRI model showed improved predictive probability. Overall, the present study demonstrated that low GNRI values predict early mortality in incident HD patients.

Many nutritional assessment tools have been developed to assess and monitor nutrition status in HD patients [9,17,19,20]. The Subjective Global Assessment (SGA) scale and Malnutrition–Inflammation Score (MIS) are validated nutritional screening methods that were reported to be related to morbidity, mortality, inflammation, and quality of life in patients undergoing HD [7,21,22]. However, these tools require subjective assessments. GNRI, a simple and fully objective nutritional method, was proposed for nutritional risk assessment in HD patients. Previous cross-sectional studies showed the predictive validity of GNRI for mortality in HD patients [10,11,14]. Beberashvili et al. compared the longitudinal performance of the MIS and GNRI for patients on maintenance HD [23]. They reported that both MIS and GNRI are useful tools for the longitudinal observation of the nutritional status of prevalent HD patients and should be used for this purpose. In a study of Japanese chronic HD patients, a low GNRI closely reflected the protein-energy wasting (PEW) state defined by the International Society of Renal Nutrition and Metabolism criteria, and GNRI is suitable for primary assessment of PEW because this indicator is simple and convenient [24].

Malnutrition is common and strongly related with increased mortality in patients with ESRD [25,26]. In patients with chronic kidney disease (CKD) stage 4–5, restricting protein intake (0.8 g/kg/day) is recommended to attenuate the progression of kidney disease. Therefore, the nutrition status of patients with CKD may be changed according to the dietary recommendations before starting renal replacement therapy such as HD or PD. PEW, characterized by a loss of body protein mass and energy fuel reserves, is common in patients with CKD and ESRD [27]. With a high prevalence in those patients, PEW has been associated with adverse clinical outcomes and mortality [28,29]. Therefore, assessment and monitoring of protein and energy status are essential to prevent uremic malnutrition in patients who initiate HD. However, there are few studies on the effect of nutritional status at the initiation of HD on early mortality in HD patients. This study shows that the nutrition status is an important predictive factor for early mortality, and that GNRI can be a useful tool for the assessment of nutritional status in this population.

Patients on HD have a much higher mortality rate than the general population. Although overall mortality rates of the ESRD population are improving, the period immediately after the transition to ESRD is associated with the highest mortality rates. Mortality rates up to 30% have been described within the first year of transition from CKD to ESRD [30,31,32]. Understanding patient circumstances during the early ESRD period may help improve survival [33]. Thus, attempts to improve HD mortality outcomes in this period are needed in patients who initiate HD. Several studies have analyzed the factors that can influence this increased early mortality. Among them are nonmodifiable factors such as age, sex, and previous pathologies, and modifiable factors such as malnutrition, vascular calcification, anemia, and the type of vascular access [1,34,35,36]. Malnutrition is an important and modifiable factor for mortality in incident HD patients. Therefore, clinicians should be alerted to assessing the nutritional status in those patients, and a simple and reliable nutritional assessment tool can be useful. 

In this study, the predictive probability of GNRI for all-cause mortality was better than that of BMI or serum albumin during early HD treatment. It has been reported that hypoalbuminemia and low BMI could be an important indicator of malnutrition [37]. Hypoalbuminemia is common in HD patients and associated with mortality [38]. Because albumin is a negative acute phase reactant and levels decrease in patients with inflammation, hypoalbuminemia can be a result of malnutrition or simply a reflection of inflammatory states [38,39]. In a recent meta-analysis, high BMI was associated with a lower all-cause mortality rate in HD patients [40]. Malnutrition in the ESRD population is a complex syndrome that is affected by multiple factors. Because GNRI might integrate these factors, it can be a useful indicator of the individual’s nutrition status and prognostic factor for mortality. 

There are several limitations to this study. First, our study is an observational study and included only the Korean population. Further studies should be performed to confirm the predictive value of GNRI for early mortality in incident HD patients in other populations. Second, patients in Quartile 4 were significantly younger than those in Quartile 1. We adjusted for age in the multivariate analysis, but due to the large difference in age between each group, we performed an additional stratification analysis based on an age of 65 years. The results showed that there was no difference in the direction of the outcomes in each group based on age. Age did not significantly affect the outcomes (*p*-value for interaction = 0.409). Third, the GNRI calculation includes albumin, which is affected by non-nutritional factors, such as inflammation. Unfortunately, our data did not include information on C-reactive protein, which could have helped us account for inflammation. The albumin level might decrease in the volume overload status. In addition, this study did not estimate fragility syndromes, which may affect mortality. Despite this limitation, this is one of the largest observational studies of incident HD patients investigating the association between low GNRI and mortality in the first year after the initiation of HD.

## 5. Conclusions

In conclusion, our study demonstrates that a GNRI < 91.8 is associated with all-cause and cardiovascular mortality in HD patients during the early ESRD period. GNRI is a measure that physicians can easily calculate in patients who have started HD and can be used for convenient prognostic purposes. These findings suggest that GNRI is a significant predictor of mortality in these patients.

## Figures and Tables

**Table 1:** Baseline characteristics of the patients by GNRI quartiles, Korea Renal Data System, 2016–2019.

	Total (*n* = 10,545)	Quartile 1 56.7–91.7 (*n* = 2637)	Quartile 2 91.8–97.1 (*n* = 2638)	Quartile 3 97.2–101.2 (*n* = 2637)	Quartile 4 101.3–129.6 (*n* = 2633)	*p*-Value
Age at first HD, year	63.9 ± 13.7	68.0 ± 13.4	65.1 ± 13.3	63.1 ± 13.1	59.5 ± 13.4	<0.001
Women	3919 (37.2)	1077 (40.8)	1081 (41.0)	974 (36.9)	787 (29.9)	<0.001
Main cause of ESRD						<0.001
Diabetes	5587 (53.0)	1317 (49.9)	1418 (53.8)	1461 (55.4)	1391 (52.8)	
Hypertension	2143 (20.3)	514 (19.5)	552 (21.0)	525 (19.9)	550 (20.9)	
Glomerulonephritis	735 (7.0)	177 (6.7)	175 (6.8)	181 (6.9)	198 (7.5)	
Others	2080 (19.7)	629 (23.9)	480 (18.5)	470 (17.8)	494 (18.8)	
SBP (mmHg)	143.2 ± 19.5	141.5 ± 20.6	143.2 ± 20.0	144.0 ± 19.1	144.0 ± 18.4	<0.001
DBP (mmHg)	76.0 ± 12.4	74.6 ± 12.9	75.8 ± 12.5	75.9 ± 11.9	77.7 ± 12.1	<0.001
Body mass index (kg/m^2^)	22.8 ± 3.7	20.6 ± 3.4	22.8 ± 3.6	23.8 ± 3.4	24.2 ± 3.4	<0.001
Kt/V	1.48 ± 0.28	1.49 ± 0.30	1.50 ± 0.29	1.47 ± 0.28	1.44 ± 0.26	<0.001
Comorbid conditions						
Coronary artery disease	1012 (9.6)	254 (9.6)	265 (10.0)	247 (9.4)	246 (9.3)	0.808
Heart failure	513 (4.9)	185 (7.0)	113 (4.3)	118 (4.5)	97 (3.7)	<0.001
Arrhythmia	302 (2.9)	92 (3.5)	78 (3.0)	77 (2.9)	55 (2.1)	0.023
Cerebrovascular disease	848 (8.0)	2789 (10.5)	206 (7.8)	189 (7.2)	175 (6.6)	<0.001
COPD	74 (0.7)	28 (1.1)	16 (0.6)	18 (0.7)	12 (0.5)	0.056
Liver disease	415 (3.9)	146 (5.5)	108 (4.1)	78 (3.0)	83 (3.2)	<0.001
GI disease	1116 (10.6)	309 (11.7)	277 (10.5)	270 (10.2)	260 (9.9)	0.149
Vascular access						<0.001
Fistula	7017 (66.5)	1250 (47.4)	1727 (65.5)	1962 (74.4)	2078 (78.9)	
Graft	1665 (15.8)	445 (16.9)	451 (17.1)	404 (15.3)	365 (13.9)	
Catheter	1863 (17.7)	942 (35.7)	460 (17.4)	271 (10.3)	190 (7.2)	
Hemoglobin, g/dL	10.4 ± 1.2	9.9 ± 1.4	10.4 ± 1.2	10.5 ± 1.0	10.7± 1.0	<0.001
Albumin, g/dL	3.8 ± 0.5	3.2 ± 0.4	3.7 ± 0.2	4.0 ± 0.1	4.3 ± 0.2	<0.001
Calcium, mg/dL	8.5 ± 0.8	8.3 ± 0.9	8.5 ± 0.	8.6 ± 0.7	8.8 ± 0.8	<0.001
Phosphorus, mg/dL	4.7 ± 1.4	4.2 ± 1.4	4.7 ± 1.4	4.8 ± 1.4	5.2 ± 1.4	<0.001
Intact PTH, pg/mL	197.0 ± 176.3	179.4 ± 167.6	204.3 ± 199.5	200.0 ± 172.6	204.4 ± 162.2	<0.001
Cholesterol, mg/dL	139.7 ± 38.4	139.6 ± 41.2	139.9 ± 37.5	140.0 ± 37.2	139.4 ± 37.7	0.956

Data are number (percentage) and mean ± standard deviation. Abbreviations: HD, hemodialysis; ESRD, end-stage renal disease; SBP, systolic blood pressure; DBP, diastolic blood pressure; COPD, chronic obstructive pulmonary disease; GI, gastrointestinal; GNRI, Geriatric Nutritional Risk Index; PTH, parathyroid hormone.

**Table 2:** Cox proportional Hazard Ratios (95% Confidence Intervals) for first-year mortality.

	Univariate	*p*-Value	Multivariate	*p*-Value
All-cause				
GNRI quartile				
Quartile 1	7.74 (5.76–10.36)	<0.001	2.56 (2.13–3.09)	<0.001
Quartile 2	2.08 (1.48–2.91)	<0.001	1.33 (1.06–1.68)	0.016
Quartile 3	1.28 (0.89–1.86)	0.18	1.00 (0.76–1.32)	0.997
Quartile 4	Reference		Reference	
Cardiovascular				
GNRI quartile				
Quartile 1	6.22 (4.07–9.50)	<0.001	2.29 (1.71–3.08)	<0.001
Quartile 2	1.51 (0.91–2.50)	0.11	1.05 (0.72–1.53)	0.804
Quartile 3	0.92 (0.52–1.61)	0.77	0.75 (0.48–1.17)	0.205
Quartile 4	Reference		Reference	

Abbreviation: GNRI, Geriatric Nutritional Risk Index. Multivariate model: adjusted for age, sex, main cause of ESRD, systolic blood pressure, diastolic blood pressure, presence of comorbidities, vascular access, and laboratory findings.

**Table 3:** Comparison of predictive values for mortality.

Models	AUC (95% Confidence Intervals)	*p*-Value
All-cause		
Baseline	70.8 (68.4–73.2)	
+BMI	72.2 (69.8–74.5)	0.002
+Albumin	76.2 (73.9–78.4)	<0.001
+GNRI	77.1 (74.8–79.2)	<0.001
BMI vs. GNRI		<0.001
Albumin vs. GNRI		0.009
Cardiovascular		
Baseline	70.1 (66.4–73.8)	
+BMI	70.6 (66.9–74.4)	0.518
+Albumin	75.1 (71.4–78.7)	<0.001
+GNRI	75.7 (72.2–79.0)	<0.001
BMI vs. GNRI		<0.001
Albumin vs. GNRI		0.132

**Table 4:** Subgroup analyses for all-cause mortality.

	Hazard Ratio (95% Confidence Interval) *	
	Quartile 1 (vs. Quartile 4)	*p*-Value for Interaction
Age		
<65 years	1.91 (1.29–2.84)	0.409
≥65 years	2.50 (1.99– 3.14)	
Sex		
Woman	1.67 (1.17–2.40)	0.659
Man	3.03 (2.39–3.84)	
Diabetes		
Yes	2.00 (1.54–2.59)	0.076
No	3.48 (2.56–4.72)	
History of CVD		
Yes	3.59 (2.42–5.32)	0.185
No	2.20 (1.74–2.77)	
Vascular access		
AVF	2.59 (1.93–3.48)	0.711
AVG or catheter	2.41 (1.81–3.23)	
Hemoglobin		
<10 mg/dL	2.23 (1.62–3.09)	0.722
≥10 mg/dL	2.60 (2.00–3.37)	
Phosphorus		
<5 mg/dL	3.03 (2.39–3.83)	0.342
≥5 mg/dL	1.79 (1.18–2.72)	

Abbreviations: AVF, arteriovenous fistula; AVG, arteriovenous graft; CVD, cardiovascular disease. * Adjusted for age, sex, main cause of ESRD, systolic blood pressure, diastolic blood pressure, presence of comorbidities, vascular access, and laboratory findings.

## Data Availability

The data presented in this study are available on request from the corresponding author.
